# Badminton Players’ Moral Intentions: A Factorial Survey Study Into Personal and Contextual Determinants

**DOI:** 10.3389/fpsyg.2019.02272

**Published:** 2019-10-09

**Authors:** Els De Waegeneer, Bram Constandt, Stef Van Der Hoeven, Annick Willem

**Affiliations:** Department of Movement and Sports Sciences, Ghent University, Ghent, Belgium

**Keywords:** badminton, factorial survey, fair play, moral intention, sports ethics

## Abstract

Improving and maintaining high ethical standards among athletes is a major challenge in sports, which requires sufficient knowledge on athletes’ moral intentions. This study advances our knowledge on athletes’ moral intentions by examining the personal and contextual determinants (factors) that influence moral intentions of badminton players. In a factorial survey study, a total of 171 participants were asked to respond to scenarios describing moral dilemmas in the context of badminton. This approach allows combining advantages from both classical experiments and survey methods, enabling the determination of the underlying principles of the judgments and intentions of respondents. Multilevel analysis indicated that intention to engage in the described behavior was impacted by both the act and the gender of the subject. This study complements previous research on athletes’ moral intentions by the advanced method of factorial survey, while supporting the development of more specific approaches in the promotion of ethical behavior in sports.

## Introduction

The call for more ethical behavior in sports has become more manifest in recent years. Major sports organizations, as well as other actors – such as UNESCO – are increasingly expressing their concerns about misbehavior in sports (Vanden Auweele et al., 2016). Moreover, (the lack of) ethical behavior in sports has been the subject of an increasing body of literature, and has given rise to an ongoing public and academic debate (Kavussanu and Stanger, 2017). A consensus in this debate is that the challenges in sports are very diverse, ranging from the use of performance-enhancing substances (see, e.g., Pitsch and Emrich, 2012; Morente-Sánchez and Zabala, 2013), over gender inequality (see, e.g., Flake et al., 2012; Lagaert and Roose, 2018), to match-fixing (see, e.g., Chappelet, 2015; Tak, 2018).

A quintessential concept that is often used in the debate about the ethical challenges in sports, is Fair Play. Fair Play can be defined in different ways, such as “behaving according to the rules” or “conduct in agreement with the spirit of the game” (Sheridan, 2003; Boixadós et al., 2004; De Waegeneer and Willem, 2016). The most interesting situations, regardless of the used definition, occur when athletes enter the “gray zone,” referring to situations in which an opportunity exists to engage in questionable behavior to win the game. This kind of behavior can vary from “raw cheating” (e.g., lying about the position of an offside ball or shuttle), over an intimidating style of playing, to engaging in very strategic ways to play the game (e.g., “tanking” or “sandbagging,” which refers to underperforming, or losing deliberately to face a supposedly weaker opponent in the following competition) (Sailors et al., 2015).

All the aforementioned examples occur on the sports fields, and raise questions concerning the ethical soundness of the reported behaviors (Duda et al., 1991; Shields et al., 2007). However, scant understanding exists about (a) how ethical behavior can be encouraged in these situations, and (b) which determinants (factors/characteristics) influence the intentions to engage in these behaviors (Brunelle et al., 2005; Miller et al., 2005; Kavussanu and Spray, 2006; Shields et al., 2007; Sailors et al., 2015). So far, there has been little research on the intentions of athletes to come to ethical behavior, neither on the elements that are crucial in this decision-making process. Given the sensitivity of the subject, there is a need for an instrument that assesses these intentions without losing validity to the expected social desirability bias. As a consequence, this present study empirically tests which determinants influence the intentions of athletes to engage in questionable behavior, on a large scale, and by using a solid measure – the factorial survey research design – that can withstand this social desirability trap.

Guided by the abovementioned context, the outline of this study is as follows. First, the literature review of this study discusses the concept of ethical behavior and intentions, and the approach to come to ethical behavior in a sports setting. Moreover, it presents the possible determinants of moral intentions on the sports field. Second, the methodology section explains the factorial survey method, which is applied to study the occurrence of these determinants in athletes. After the presentation of our findings in a following section, the discussion section concludes with (a) highlighting the limitations of this study and (b) explaining the opportunities that the findings of this study can bring to current research in sports ethics.

## Literature Review

### Ethical Behavior

Several authors have discussed how people come to perform in an ethical manner (O’Fallon and Butterfield, 2005). However, the predominant model of this process is put forward by Rest (1986). Although this model has a certain age, this extensive work on the understanding of ethical behavior remains today’s benchmark for studying ethical behavior (Romand et al., 2009; Rudd et al., 2010; Craft, 2013; Constandt et al., 2018). According to Rest (1986), any form of ethical behavior can be broken down into a four-steps process. A persons needs to complete these steps to establish ethical behavior: moral sensitivity, moral judgment, moral motivation, and moral character. The first of these essential steps is moral sensitivity or moral awareness, and has to do with one’s capability to interpret a situation and recognize the moral issues that are related to it (Kavussanu and Spray, 2006; Lincoln and Holmes, 2011). Second, moral judgment is the cognitive process an individual undergoes to make a moral evaluation of the issue at stake (Constandt and Willem, 2019). A third component is the intention to prioritize the value of morality over other values or motives, such as power and monetary gain (De Waegeneer and Willem, 2016). This step is called moral motivation or moral intention (Kavussanu and Spray, 2006). The final step is moral character, which is one’s capability to overcome resistance and fatigue in order to translate the moral intention into actual practice (Kavussanu and Spray, 2006). All steps need to be successfully taken to come to ethical practices or behavior (Romand et al., 2009; Lincoln and Holmes, 2011; Constandt and Willem, 2019).

This framework can also be applied to the sports context (Bredemeier and Shields, 1995; Miller et al., 2005; Romand et al., 2009; Constandt et al., 2018; Constandt and Willem, 2019). For instance, the issue of taking or leaving doping, can be broken down into these four steps: the first step would be that the athlete is aware of the ethical challenge that comes with taking doping. The second step would be to make the judgment whether doping is morally wrong or not. This is followed by the moral motivation/intention to dope or not, given the importance of playing “clean” versus the importance of winning or other interests (money, reputation, etc.). In the final step, the moral intention is converted into the resulting ethical behavior (doping or not).

The third step, moral motivation or moral intention, is a crucial element in the ethical behavior of athletes. It is a basis for (future) ethical behavior of athletes, as the literature shows a high correlation between the intended behavior and the actual behavior in sports settings (Kavussanu and Spray, 2006; Romand et al., 2009; Kavussanu and Stanger, 2017). Furthermore, research demonstrates a strong positive relationship between reported and observed unethical (i.e., questionable) behavior (Kavussanu et al., 2006). Athletes that indicate intentions to engage in certain (questionable) behavior are more likely to perform the actual behavior, as put forward by the model of Rest (1986).

### Determinants of Moral Intentions

The question remains which determinants influence the intentions that people develop on the sports field. A thorough search in the existing academic literature has led us to an inventory of possible determinants of moral intentions (Stephens et al., 1997; Tod and Hodge, 2001; Gardner and Janelle, 2002; Ommundsen et al., 2003; Brunelle et al., 2005; Miller et al., 2005; Shields et al., 2007). Both personal and contextual variables are supported by the literature as being influential to moral intentions and moral behavior. For instance, Shields et al. (2007) have taken a look at which personal attitudes predict sportspersonship, whereas Miller et al. (2005) and Kavussanu and Spray (2006) have investigated the influence of the surrounding factors, such as spectator behavior.

Looking at personal determinants that are discussed and studied in the literature, the gender, competition level, and age of the respondent are some of the most likely variables to have an influence on their moral intentions (Miller et al., 2005; Shields et al., 2007; Romand et al., 2009; Mouratidou, 2017). The importance of gender differences has been identified in previous research (Sage and Kavussanu, 2007; Steinfeldt et al., 2011). In particular, it has been indicated that males report more unsportpersonship behavior compared to females (see Shields et al., 2007), while males also score higher in terms of aggressive tendencies (see Bredemeier, 1985). This is consistent with the findings that males are more likely to approach injurious behavior and aggressions as legitimate than female (Bredemeier and Shields, 1986; Gardner and Janelle, 2002). Moreover, Guivernau and Duda (2002) have also indicated that males are more inclined to cheat than females, at a point when the game or match might be lost. Finally, gender role conflict – tensions as a consequence of dominant gender role expectations – has been indicated as another potential determinant of moral functioning (Steinfeldt et al., 2011).

Next to gender, age is also considered as a possible determinant (Ebbeck and Gibbons, 2003; Romand et al., 2009; Mouratidou, 2017). For example, Ebbeck and Gibbons (2003) and Higgins et al. (1984) have suggested that people proceed through several moral development phases during their life, and that some moral competencies are only established when people reach a certain age or maturity. Accordingly, the age of the subject should be examined as a possible determinant of moral intentions (Ebbeck and Gibbons, 2003).

A final personal determinant is the competition level of the respondent. The competition level of the subject, namely whether the person judging plays sports on a recreational or competition level could also be a possible determinant of ethical behavior (De Waegeneer and Willem, 2016; Mouratidou, 2017). Unethical behavior in sports appears to be more frequent as the competition level of the subject increases (Shields et al., 2007). It is a factor that should be considered as well when it comes to moral intentions.

Besides the aforementioned personal determinants, contextual influences on the moral intention of athletes should also be investigated. Ethical behavior takes place in a social context, which is assumed to be of great influence on the intentions and actual behavior of a person (Stephens et al., 1997; Kavussanu and Spray, 2006). Studies on Fair Play and ethical behavior in the sports context have investigated different forms of (possible) violations, such as cheating, aggression, and injury faking (Stephens et al., 1997; Tod and Hodge, 2001; Ommundsen et al., 2003; Brunelle et al., 2005; Miller et al., 2005). As such, it is important to investigate whether certain acts are more likely to be conducted by the athletes, such as cheating or aggression, rather than assuming that an athlete always reacts in the same way, according to the same moral intention.

Another contextual variable is the level of the match. Research shows that different behaviors were manifested when more was at stake during a given match. Thus, it is no surprise that Miller et al. (2005) have described that there is a positive link between contexts with a dominant focus on winning and performing and lower scores on moral judgment, next to a positive relation between high performance contexts and the legitimatization of using intimidation in sports. Thus, as it could be possible that subjects have distinct (or less) moral intentions when more is at stake, the determinant “level of the match” was included.

Furthermore, Tod and Hodge (2001) have also pinpointed to the important impact of significant others in ethical behavior. In the sports context, an important role concerning ethical behavior is given to the referee. The judgment of this person could be key in the moral intentions and the resulting ethical behavior of the athlete. The role of the referee will be included as a possible determinant of ethical behavior in the survey. The context described above does also involve the spectators that react to certain acts during a match. Spectator behavior was regarded as an important element in ethical decision-making by Shields et al. (2007). The reaction of the public can have consequences for the moral intentions, and ethical behavior of the athletes.

A final possible contextual determinant is the presence of ethical guidelines (Kaptein and Schwartz, 2008). The explicit mentioning of guidelines could influence the ethical decision-making of the respondent, by emphasizing the importance of ethical behavior in the situation. Ethical guidelines mostly take the form of concrete behavioral rules. Within organizations, these guidelines are often included in a broad ethical code.

In sum, our research objective is to assess the moral intentions of athletes, namely to investigate whether athletes are intended to engage in questionable behavior. Gaining insights in this regard is important to improve the ethical standards among athletes. The focus is put exclusively on badminton athletes, as “sandbagging” (i.e., the act of deliberately under-performing) is quite common – and often sanctioned – in this specific sport (Sailors et al., 2015). Previous research has shed light on the moral judgment of badminton athletes, but we remain largely in the dark regarding their moral intentions (De Waegeneer and Willem, 2016). The novelty of this present study resides in the examination of the role of different personal and contextual determinants (and their combinations) on the reported intention of the athlete. Some variables were studied earlier, but the methods used in previous studies could not exclude a great deal of social desirability bias. Moreover, previous studies did not combine the different possible variables in one study. The Factorial Study approach contributes to the understanding of the influences of the different variables on moral intention, and due to its particular design, this method allows to overcome social desirability bias.

## Methodology

### Sample

This study is part of a broader project that has been approved by the independent commission for medical ethics of Ghent University (De Waegeneer and Willem, 2016). To realize a diverse set of respondents in terms of their experience and level of play, data were collected at badminton tournaments at different competition levels in Flanders. Additionally, the questionnaire was dispersed via the Flemish Badminton Federation, and via the Facebook pages of the Flemish badminton clubs. Informed written consent was received from all participants, while anonymity was guaranteed to stimulate reliable answers. Moreover, participants were given the option to pose questions or to give feedback on the questionnaire. Tournaments on different levels of play were incorporated during the data collection, which leads to a reduced chance of selection bias. Moreover, the online invitations of the Flemish Badminton Federation and on Facebook were open to everyone. As such, all ages (14–65), genders, and levels of expertise were present in the sample.

A total of 171 participants were included in the final sample (73.4% males; 50.3% between 26 and 49 years of age; 82.8% were competitive players) (De Waegeneer and Willem, 2016). Table 1 shows the demographic characteristics of this group. Most respondents are male, but this reflects the current distribution of gender (61% males versus 39% females) in Flemish badminton clubs, as reported on the website of the Flemish Badminton Federation. The same sample was used to study badminton players’ moral judgments in previous work (De Waegeneer and Willem, 2016). However, as outlined below, different methodological instruments (i.e., other vignettes) were implemented to consider how the determinants of moral intentions might differ from those of moral judgments.

**TABLE 1:** Sociodemographic characteristics of respondents.

***N* = 171**	
**Sex (%)**	
Male	73.4
Female	26.6
**Age group (%)**	
14–18	11.4
19–25	32.3
26–49	50.3
50–62	6.0
**Competition level of the subject (%)**	
Recreational	17.2
Competition	82.8

### Factorial Survey Study

The factorial survey approach is applied to our study. This is an advanced method to measure the beliefs, attitudes, and judgments of respondents (Atzmüller and Steiner, 2010). In this research design, carefully constructed, realistic case descriptions, in the form of vignettes, are presented to respondents to enable a judgment about a realistic scenario (Taylor, 2006). This offers the technique high external validity. In our study, the participants were badminton players, the judgments were normative, and the scenarios occurred in a sports context the participant was familiar with. Using sentences in a fixed order, the vignettes describe a scenario that contains factors (such as the level of the game in our case) that are relevant to the normative judgment (Taylor, 2006). The relevant factors and their factor levels have to be determined according to a systematic literature review and based on a strong theoretical framework (Brauer et al., 2009; Atzmüller and Steiner, 2010). The factor is randomly included throughout the vignettes (De Waegeneer and Willem, 2016). After this, a randomly selected unique set of vignettes will be presented to the different respondents to express their judgment. Doing so, the influence of multiple factors in complex decisions on fair play in sport can be examined (De Waegeneer and Willem, 2016). The randomization that was applied to the scenarios and to the allocation of vignettes to the participants, stimulates the robustness of our methodological approach (Taylor, 2006). Furthermore, the robustness of the statistical analysis is increased by using the vignette as the unit of analysis resulting in a large sample (Taylor, 2006).

The factorial survey method has many advantages to offer. First of all, in comparison to traditional survey items, several explanatory and contextual factors can be presented at the same time, leading to more realistic scenarios (Atzmüller and Steiner, 2010). The short scenarios can be constructed with the specific target audience (in this study: badminton players) in mind (Ashill and Yavas, 2006). As such, the scenarios are fictive yet realistic, and adapted to the actual contexts in which ethical dilemmas in badminton occur (De Waegeneer and Willem, 2016). The method is also less subject to social desirability bias since respondents are less aware of the controlled variation, i.e., the factor that is examined, in comparison to conventional survey items (Alexander and Becker, 1978). A precise assessment of each variable is allowed thanks to the possible systematic variations of the characteristics used in the vignettes (Ashill and Yavas, 2006; Wallander, 2009). Another advantage of this design is the greater involvement with the respondents (Fredrickson, 1986). A potentially sensitive topic can be discussed more freely, as it can be considered more as a realistic story, rather than as a personal experience, and could be less threatening for the respondents (Barter and Renold, 1999), probably because they feel less accountable for their decisions and judgments (Taylor, 2006). An additional advantage is that the respondents’ requirement to integrate their own contextual information – leading to more biased responses – is reduced using scenarios (Fredrickson, 1986; Wallander, 2009; De Waegeneer and Willem, 2016).

A vignette universe has been constructed with the different factors (so-called “dimensions”), and levels (values for that dimension). Literature suggests that factorial survey designs preferably include five to ten factors, and the same number of factor levels for each factor (Atzmüller and Steiner, 2010). In our study, this preference is met, as there are five factors with each two or three levels. This gathered a total of 162 vignettes. To get valid results, each vignette had to be rated by at least five respondents. According to the literature, a deck of six vignettes for each respondent is suited, as it is important to avoid fatigue effects in the respondents when presenting too much vignettes (Atzmüller and Steiner, 2010). This leads to the requirement that at least 135 respondents need to participate in our study in order to find robust findings.

### Vignettes

To be able to write the vignettes with determinants adjusted to the specific context of badminton, a focus group was conducted with people having extensive experience in the field of badminton, such as members from the Flemish Badminton Federation, academic coaches in badminton, recreational and competition athletes, and parents of elite players. The focus groups was put together based on the practical suggestions of Krueger and Casey (2015). The focus group was carefully planned. Participants were meticulously selected, using their relevant expertise regarding the topic at study as the most important inclusion criterion (Krueger and Casey, 2015). Also, a moderator was present to enable the main researcher to focus exclusively on the content of the conversation (Krueger and Casey, 2015).

One of the determinants that needed to be translated by the focus group to the badminton context was the variable “Act.” Three different, relevant acts were formulated: (a) losing deliberately to meet a supposedly weaker opponent in the following competition (i.e., deliberately under-performing or “sandbagging,” see Sailors et al., 2015), (b) name-calling the opponent, and (c) not mentioning the shuttle was out when the opponent was not able to see this. The other possible determinants of moral intentions are the independent variables in our study: competition level of subject, gender of the subject, level of the match, age of subject, role of the referee, role of the audience, and presence of ethical guidelines. Each of these levels were determined for the specific badminton context. Table 2 displays the vignette universe applied to the specific context of a badminton game with the different factors or “dimensions,” and levels or “values for that dimension.”

**TABLE 2:** Different dimensions and levels of the vignettes in the vignette universe.

**Dimension**	**Different levels of the dimension**
Act	Losing deliberately, to face a weaker opponent in the following competition round
	Verbal aggression
	Not reporting the shuttle was out, when it fell on the line
Level of the match	Recreational
	National tournament
	Olympic Games
Presence of ethical guidelines for the players	(blank)
	An ethical code that states Fair Play is present
Reaction of the referee	(blank)
	In favor of the player
	In favor of the opponent
Reaction of the public	(blank)
	In favor of the player
	In favor of the opponent

The questions asked to the respondents, after the presentation of each vignette, measuring intentions was: “Would you engage in this behavior yourself?.” The outcome variable was questioned on a five point Likert scale. Gaining knowledge on athletes’ moral intentions is important in unraveling which moral decision making steps are at risk to come to ethical behavior.

**FIGURE 1:**
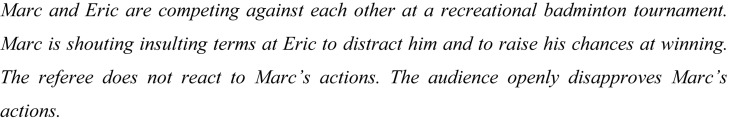
Example of a vignette.

After the development phase of the vignettes, the vignettes were again presented to a focus group of methodologists, specialized in Factorial Survey Studies. Subsequently, the sampling of the vignettes was then executed randomly, yet with stratification for the dimension “Act.” This was done each time so that each respondent gets to answer two questions about the three different acts in their personal deck of vignettes. The CAWI method, computer-assisted web interview, was used to integrate these vignettes into questionnaires, after the sampling. The program “Qualtrics” was used to integrate the so-called decks (i.e., the groups of vignettes to show to the respondents). These decks were sampled randomly with replacement, so that every deck was returned in the universe after its withdrawal (De Waegeneer and Willem, 2016). To prevent methodological artifacts caused by the specific order of the vignettes, a random presentation order of the vignettes to the respondents was integrated. In addition to the vignettes, respondents were requested to fill in their age, gender, club, and competition level. An example of a vignette is given in Figure 1.

### Data Analysis

As was also done in previous work (see De Waegeneer and Willem, 2016), the completed questionnaires were analyzed using descriptive statistics and mixed model statistical techniques, using SPSS 22 software. Multilevel models were constructed to respect the variation of vignette characteristics (level one – i.e., act, level of the match, reaction of the referee, reaction of the audience, and presence of ethical guidelines), and for respondent characteristics (level two – i.e., age of the subject, gender of the subject, competition level of the subject).

## Findings

Table 3 showcases the findings of the three different multilevel models in their order of development. Findings indicate that model 1 was significant.

**TABLE 3:** Significant effects of the vignette and respondent characteristics on the intention to engage in the questionable behavior.

	**Intention to engage in this behavior (= moral motivation)**		
**Fixed effects**	**Model 1**	**Model 2**	**Model 3**

Constant *(SE)*	1.934 (0.060)^∗^	1.544 (0.073)^∗^	1.517 (0.121)^∗^
**Level 1**			

**Act**			
(ref. shuttle out)			
Losing deliberately		0.929^∗^	0.929^∗^
Verbal aggression		0.240^∗^	0.240^∗^
**Level of the match**			
(ref. Olympic)			
Recreational		−0.120	−0.120
National		0.064	
**Presence of guidelines**			
(ref: present)		0.018	0.018
**Reaction referee**			
(ref: blank)			
Opponent		0.030	0.030
Player		−0.080	−0.080
**Reaction public**			
(ref: blank)			
Opponent		0.010	0.010
Player		−0.012	−0.012

**Level 2**			

**Gender of the subject**			0.364^∗^
(ref. male)			
**Age**			
(ref: 50–62)			
14–18			0.067
19–25			0.135
26–49			0.227
**Competition level**			0.094
(ref: recreational)			
−2 Log Likelihood		3016.306	3011.150
Δ 2 Log Likelihood		281,746	286,902
	3298.052		

*^∗^*p* < 0.05.*

Model 1: This model has no level one or level two predictors.
Model 2: This model includes all predictors on the first level (vignettes), assesses the effect of vignette characteristics, namely act, level of the match, reaction of the referee, reaction of the audience, and presence of ethical guidelines, on the dependent variables. Comparing this model with the null model shows that the variable “Act” (level one) contributes significantly to the intention to engage in the proposed behavior. 17.0% of the variance in the scores on the inclination to engage in the behavior is explained by “Act.” Other variables on this first level appear to be not significant. Neither the level of the match, the presence of ethical guidelines nor the reaction of the referee or the audience was able to change the intention of the athletes.
Model 3: included the second level variable, namely: the respondent characteristics, namely age of the subject, gender of the subject, and competition level of the subject. The findings in the third model state that the variable “Gender of the subject” (level two) contributes significantly to the intention to engage in the proposed behavior. 5.0% of the variance in the scores on the inclination to engage in the behavior is explained by the “Gender of the subject.” Other variables on this level, that appeared important in literature, such as the age and the competition level of the subject, did not significantly contribute to the intention to engage in the questionable behavior.

## Discussion

Ethical behavior is the final result of a four-step process (Rest, 1986), that involves (a) the awareness of the ethical issue at hand, this is the moral sensitivity; (b) the careful deliberation of the issue, which leads to the moral judgment; (c) the intention to live up to this judgment, i.e., the so-called moral motivation and finally; and (d) the actual behavior, i.e., the moral character. This study focuses on the third step in the process, namely the moral intention, and therefore examines the intention of athletes to engage in ethical behavior when winning is at stake. Research showed a strong positive relationship between reported and observed unethical/questionable behavior (Kavussanu et al., 2006).

While investigating the intentions of badminton players, our research also complements existing studies by drawing on a quasi-experimental method that measures the personal and contextual determinants that influence the intentions of the athletes. Due to the advanced factorial survey approach, the social desirability bias is greatly diminished, as respondents are less aware of the controlled variation of the different elements in the vignettes (Wallander, 2009) and therefore a light can be shed on what actually influences the moral motivation of athletes. These influencing variables are important knowledge in attempts to promote ethical behavior.

When looking at the personal determinants that influence the intentions of athletes to act in an ethical manner, our findings show that the gender of the subject is an important variable. The findings point out that men are more intended to engage in questionable behavior than women. This result is in agreement with earlier research on ethical behavior in sports, which have consistently reported on gender differences, such as the study of Guivernau and Duda (2002) that identified males as more accepting of cheating compared to females when losing was near, and the study of Ebbeck and Gibbons (2003). This is also consistent with Shields et al. (2007) who provide evidence for the fact that males engage more in “unsportspersonship” behavior than females. Sage and Kavussanu (2007) confirm in their study that females displayed more ethical behavior. However, this finding is not in line with the work of Mouratidou (2017), who states that the gender of volleyball, soccer, basketball, and handball players does not have an effect on their moral competence. The occurrence of these mixed findings regarding the role of gender could be due to the different applied methods, and different targeted steps of morality, ranging from analyzing moral competence (i.e., moral judgment), versus assessing actual ethical behavior (i.e., moral character).

A possible explanation for the here reported gender difference is that males are more ego oriented than females (Duda et al., 1991; Sage and Kavussanu, 2007; Shields et al., 2007). Ego orientation involves a focus toward achieving a positive evaluation of their abilities and importance from others, whereas task orientation focuses on learning and improving abilities (Li et al., 1996). These different definitions of goals, and therefore success, could explain that men are more likely to “endorse doing anything that is necessary to obtain victory and display superiority” (Duda et al., 1991, p. 84), as they are more directed toward winning. Moral concerns become subordinate to the desire to win and feel superior (Shields et al., 2007). This implicates that another approach might by necessary when raising awareness on the subject of Fair Play in males and females. The awarding of a Fair Play trophy, for instance, might turn ethical behavior into an ego task in itself.

Contrary to the findings of the study of Mouratidou (2017), our study did not find the age of the subject to have an influence on the intentions of the athletes to engage in the proposed behavior. Most participants were adults (88.6%) and their age categories did not give significantly different findings. Neither did the findings vary in a significant way when comparing them to the responses of adolescents (11.4% of all participants). The displayed moral competencies did not seem to differ according to age, keeping in mind that all the participants were older than 14 years. This could be different when the intentions of younger children are subjected to investigation, but this is outside the scope of our research. As such, age remains an interesting determinant to consider when studying moral development and decision making in sports. As a consequence, the discrepancy with Mouratidou’s (2017) findings could be due to the fact that our study did target universal steps to come to ethical behavior (based on the model of Rest, 1986), instead of age-bound stages of moral development (based on the model of Kohlberg, 1984). In sum, it appears that all respondents shared the same level of moral “maturity,” which implicates that age should not be something to focus on when trying to promote ethical behavior in sports.

A variable that was surprisingly of no influence on the intentions of athletes to engage in questionable behavior, is the competition level of the athlete. In contrast to the work of Shields et al. (2007) in other sports disciplines, our study showed no significant evidence for more unethical behavior according to the competition level of the subject. However, in the case of badminton, it seems of no importance in the moral motivation of athletes whether they play at the recreational or the (high) competitive level. Thus in other words, the specific context of badminton might play a role in this regard. In sports in which strong differences are present between levels of play (e.g., football/soccer and the enormous financial incentives that are present on the highest competition levels), competition level might influence moral intentions.

Next to the influence of the personal variables of the athletes on their intentions, also the contextual variables were put to the test. First of all, the act itself showed to make a significant difference. Although earlier work has investigated one or more of these acts, there is no study yet that examines the understanding of these acts toward one another. The order of these acts in relation to athletes’ intentions showed that they are more likely to conduct “strategic” questionable behavior than verbal aggression or “raw” cheating. They are inclined to lose deliberately to meet a supposedly weaker opponent in the following competition round, which makes us conclude that they do not consider this unethical behavior or that they are still willing to impose this behavior, because what is at stake (winning) prevails over moral concerns. Name-calling the opponent is intended less frequently, and even less athletes have the intention to lie about the fact that the shuttle was out when the opponent was not able to see this.

These findings have implications for the promotion of ethical behavior in sports. It shows us that more emphasis needs to be put on the gray zone of “strategic” forms of unethical behavior. If the sports world wants to ban acts, such as losing deliberately to meet a supposedly weaker opponent in the following competition round, it needs to take a clear stand on their moral status, to make sure that athletes consider this as unethical behavior as well (Sailors et al., 2015). If not, the athletes might understandably not judge these acts as unfair, but rather apply them as strategic methods to win in an ethical legitimate manner. Non-physical forms of aggression need to be tackled as well. This type of unethical behavior is not so striking or noticeable as physical aggression or “raw” cheating, and therefore, could be more legitimized by athletes. However, these acts need to be decreased as well if we want our sports to become more ethical.

The reaction of the referee and the presence of ethical guidelines did not influence the moral motivation of the athletes. Their intentions were not changed due to this contextual variable, which leads us to believe that the “formal” social context is less dominant in the intentions of the athletes than is considered by some authors (Higgins et al., 1984; Tod and Hodge, 2001). However, more “informal” contextual variables, for instance the leadership style of the coaches, could be of importance and should be considered in future research (Constandt et al., 2018).

A clear judgment on the fairness of certain acts is needed in order to play in an ethical manner. The referee is seen as the authority on the fairness of events (Bertman, 2007). The fact that this role is situated outside the game, makes that the consideration of fairness is not subject to social judgments, such as the reaction from the public or judgments from the athletes themselves (Bertman, 2007). If we want the referee to maintain or regain his/her function as the insurance for the fairness of the game, more emphasis should be put on the moral authority of the referee. We cannot simply assume that this person is able to guide all the immoral intentions of the athletes into more acceptable conduct, as our findings demonstrate. If we want this person to have an influence on the intentions of athletes, measures are needed to re-establish his/her power. For instance, a program to raise the respect for referees was started a few years ago, by the Australian Government. Their awareness campaign wants to promote a greater recognition and respect for the referees, as well as a better understanding of their key role in sports.

The fact that only two of the seven tested variables had an actual impact on the intentions, i.e., the moral motivation, of athletes could have to do with the fact that our method, the factorial survey approach, rules out most of the social desirable answers. Letting your intentions, or rather the responses on questions about intentions, depend on the presence of guidelines or on the reaction of the referee could be considered as the socially desired answer. However, with the factorial survey approach, the effect of this bias is mostly ruled out and more truthful answers can be obtained. Moreover, the respondent cannot tell which social answer is desired, as the different dimensions are appointed to the vignettes in a random, experimental manner.

### Limitations

The limitations of this study should be considered when interpreting findings. First, the vignettes were specifically designed to apply to the context of badminton. This is a strength as it made the questions very relevant and tailored to our tested population, but it also makes it less transferable to other sports settings. The type of sport and its unique setting could be of great importance in the moral motivation of the athletes at hand and this specificity needs to be taken into account when generalizing our findings. Another limitation is the “traditional” critique on factorial survey studies: the method remains hypothetical in nature. Although this is off course the case, the method offers a lot of advantages that outweigh this possible limitation. The strengths of an experimental design combined with the validity of a survey method make this study design a rigorous method for studying decision-making and motivation.

### Future Research

Future research should be directed toward the broadening of the scope of sport disciplines. The investigation of the moral motivation could differ in other sports, such as medium-contact or contact sports (see Mouratidou, 2017), or in sports were the referee has a stronger or weaker status. Extending the study to other populations is therefore an important next step, as failing to compete at the best of one’s abilities is present in all (types of) sports (Sailors et al., 2015). Two other elements that should be investigated as potential determinants are (a) the impact of one’s competitive experience, and (b) whether the overall moral atmosphere and motivational climate in sports organizations contribute to the individual moral motivation of athletes, and in which way this is the case (Boixadós et al., 2004; Al-Yaaribi and Kavussanu, 2018). Organizational variables could affect the intentions of athletes on a third level, next to personal and contextual variables. Another important goal for future research should be to measure how the actual behavior – the outcome of the fourth step in Rest’s model – is influenced by these personal, contextual, and organizational variables. This would enable to clarify the relationship between intended and actual behavior. In this regard, we suggest using Ajzen’s (1991) theory of planned behavior to analyze from a holistic perspective how athletes’ moral intentions lead to moral character and actual ethical behavior.

## Conclusion

Moral intention is the second last cognitive step an individual has to take to actually come to ethical behavior. Despite its importance, limited research attention has yet been devoted to the elements (determinants) that contribute positively to people’s moral intentions in sports. This present study adds both empirically and theoretically to the sports ethics literature, showcasing the effect of the act at stake and the gender of the subject on the moral intentions of a large sample of badminton players.

These findings contribute to our knowledge on moral intentions in the sports context, while they also help to develop the necessary and suitable tools to promote ethical behavior. By applying the quasi-experimental factorial survey approach, the findings of our study have great validity and robustness when it comes to resisting social desirability bias. Moreover, the findings show a meaningful comparison between intentions toward different questionable acts, whereas they also highlight the importance of gender differences in moral intentions concerning ethical behavior in a sports context.

## Data Availability Statement

The datasets generated for this study are available on request to the corresponding author.

## Ethics Statement

The studies involving human participants were reviewed and approved by the independent commission for medical ethics of the Ghent University. The participants provided their written informed consent to participate in this study.

## Author Contributions

EW contributed to the design, data collection and analysis, and wrote the first draft of the manuscript. BC critically revised the manuscript for important intellectual content and contributed to the data analysis. SH critically revised the manuscript. AW contributed to the design and critical revision of the manuscript. All authors approved the final version of the manuscript.

## Conflict of Interest

The authors declare that the research was conducted in the absence of any commercial or financial relationships that could be construed as a potential conflict of interest.
